# Nucleic Acid Immunity and DNA Damage Response: New Friends and Old Foes

**DOI:** 10.3389/fimmu.2021.660560

**Published:** 2021-04-26

**Authors:** Clara Taffoni, Alizée Steer, Johanna Marines, Hanane Chamma, Isabelle K. Vila, Nadine Laguette

**Affiliations:** ^1^ Institut de Génétique Humaine, CNRS, Université de Montpellier, Molecular Basis of Inflammation Laboratory, Montpellier, France; ^2^ Azelead, Montpellier, France

**Keywords:** cytosolic nucleic acids, DNA damage responses, inflammation, cGAS-STING, IFI16, DNA-PK, tumorigenesis

## Abstract

The maintenance of genomic stability in multicellular organisms relies on the DNA damage response (DDR). The DDR encompasses several interconnected pathways that cooperate to ensure the repair of genomic lesions. Besides their repair functions, several DDR proteins have emerged as involved in the onset of inflammatory responses. In particular, several actors of the DDR have been reported to elicit innate immune activation upon detection of cytosolic pathological nucleic acids. Conversely, pattern recognition receptors (PRRs), initially described as dedicated to the detection of cytosolic immune-stimulatory nucleic acids, have been found to regulate DDR. Thus, although initially described as operating in specific subcellular localizations, actors of the DDR and nucleic acid immune sensors may be involved in interconnected pathways, likely influencing the efficiency of one another. Within this mini review, we discuss evidences for the crosstalk between PRRs and actors of the DDR. For this purpose, we mainly focus on cyclic GMP-AMP (cGAMP) synthetase (cGAS) and Interferon Gamma Inducible Protein 16 (IFI16), as major PRRs involved in the detection of aberrant nucleic acid species, and components of the DNA-dependent protein kinase (DNA-PK) complex, involved in the repair of double strand breaks that were recently described to qualify as potential PRRs. Finally, we discuss how the crosstalk between DDR and nucleic acid-associated Interferon responses cooperate for the fine-tuning of innate immune activation, and therefore dictate pathological outcomes. Understanding the molecular determinants of such cooperation will be paramount to the design of future therapeutic approaches.

## Introduction

Innate immunity, the first line of host defense, is classically triggered in response to pathogen infection or local lesions to promote infection clearance or wound-healing processes. The activation of innate immune responses vastly relies on pattern-recognition receptors (PRRs) that detect danger associated molecular patterns (DAMPs) or pathogen-associated molecular patterns (PAMPs). Upon recognition of PAMPs or DAMPs, PRRs trigger signaling cascades leading to the production of soluble mediators, such as type I Interferons, cytokines and chemokines. Pathogen-derived nucleic acids constitute major PAMPs that are detected by a vast array of PRRs that operate in specific subcellular localizations. In recent years, self-nucleic acids, originating from replication stress (1), DNA or mitochondrial damage (2), and endogenous retroelement activation (3), have been identified as substrates for cytosolic PRRs, and are thus considered as DAMPs. Because nucleic acids are abundant in cells, the activity of PRRs engaged in their detection is regulated and compartmentalized (4). PRRs dedicated to nucleic acid detection also present substrate specificity, with subclasses dedicated to the detection of particular moieties (5).

A plethora of cytosolic nucleic acid sensors have been described to participate in triggering Interferon responses. Such receptors notably include the ubiquitous DNA-dependent activator of Interferon regulatory factors (DAI) (6), AIM2 (7, 8), Interferon gamma-inducible protein 16 (IFI16) (9), melanoma differentiation factor 5 (MDA5) (10) and retinoic acid-inducible gene (RIG-I) (10). An extensive description of the mechanism of action of the above mentioned PRRs can be found in (11). Among pathways involved in cytosolic nucleic acid detection, the Stimulator of Interferon genes (STING) protein constitutes a central signaling hub (12, 13). Initial reports indicated that STING activation requires detection of cytosolic nucleic acid species such as double strand (dsDNA), single strand (ssDNA), or RNA : DNA species (14–16) by the cyclic GMP-AMP (cGAMP) synthase (cGAS) PRR (14). The main signature of activation of this signaling pathway is the production of type I Interferons that in turn promote the production of interferon-stimulated genes (ISGs). This signaling pathway has attracted tremendous biomedical interest in recent years, notably with observation that agonists of STING can boost antitumoral immunity (17).

However, there is emerging evidence for an intricate signaling network beyond the cGAS-STING cascade, which cannot be overlooked in therapeutic strategies aiming to boost STING activation. Of particular importance is the fact that cGAS and STING have been both described as involved in genotoxic stress response and to participate to the maintenance of genomic integrity. Furthermore, the DNA-PK complex, which is best known for its function in non-homologous end-joining (NHEJ)-mediated repair of dsDNA breaks (DSB), has been shown to serve as an alternative route to stimulate type I Interferon production (18–21). In parallel, the Interferon Gamma Inducible Protein 16 (IFI16) was also reported to detect, in concert or not with cGAS, DNA damage-derived nucleic acid species (9, 22, 23), and to cooperate with DDR proteins to promote STING-dependent immune responses following genotoxic stress (22). Furthermore, cGAS and STING have been shown to control genomic stability (24, 25). Thus, the current literature highlights tight connections between DNA repair processes and nucleic acid-associated inflammatory responses. Indeed, proteins involved in the recognition of abnormal DNA, regardless of their origin, appear to possess common roles in the initiation of inflammatory responses and surveillance of genomic integrity.

In this *mini review*, we discuss this interconnection between DNA repair mechanisms and nucleic acid immunity, by focusing on the cGAS and IFI16 receptors and the way in which they control STING activation. While several DNA repair proteins have been involved in the fine tuning of inflammatory responses (22, 26), here we focus on the DNA-PK complex, responsible for NHEJ, for which a role in controlling nucleic acid-dependent inflammatory responses has been reported (26). We discuss how dissecting these signaling networks will deepen our understanding of Interferon responses, which is likely crucial to the design of therapeutic responses to pathological inflammation.

## Cytosolic Nucleic Acid Detection: STING as a Central Signaling Hub

### The cGAS-STING Pathway

**Figure 1:**
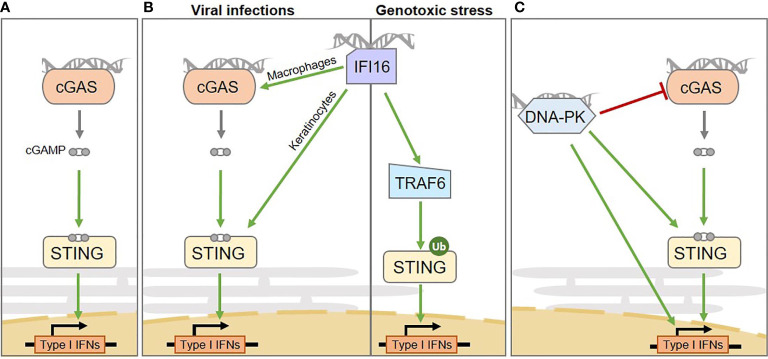
Intertwined cytosolic nucleic acid pathways involved in Interferon and pro-inflammatory cytokine production in human cells. **(A)** The cGAS sensor activates STING *via* the production of the cGAMP second messenger. **(B)** *From left to right:* the IFI16 sensor mediates inflammation through multiple routes: upon viral infection it activates STING in a cell type-specific manner, either enhancing cGAS-dependent cGAMP production in macrophages or by directly activating STING in keratinocytes; upon genotoxic stress it mediates cGAS-independent, but TRAF6-dependent STING activation. **(C)** the DNA-PK DNA repair complex was shown to play a role in inducing type I Interferon production upon cytosolic dsDNA detection. However, multiple downstream mechanisms have been proposed, that require STING activation or not, ultimately leading to the phosphorylation of transcription factors responsible for type I Interferon production. The catalytic subunit of the DNA-PK complex (DNA-PKcs) can also suppress cGAS enzymatic activity, by promoting its phosphorylation. IFNs, Interferons.

The production of type I Interferons, in the presence of cytosolic nucleic acid species, was initially described to rely mostly on cGAS (14). Indeed, cGAS detects dsDNA, ssDNA, or RNA : DNA species (14–16) in the cytosol and catalyzes the synthesis of cGAMP (**Figure 1A**). Although the binding of cGAS to nucleic acid species is sequence-independent, cGAS activation is increased by longer dsDNA fragments (27, 28), suggesting that portions of chromosomes, such as those arising in the micronucleation process, would serve as potent substrates for cGAS. cGAMP interacts with the endoplasmic reticulum (ER)-resident STING adaptor protein (29, 30), promoting conformational changes (29, 31), oligomerization (32) and translocation to perinuclear compartments, including the Golgi apparatus (12, 33). Subsequent recruitment of the TANK-binding kinase 1 (TBK1) (34), together with transcription factors, such as Nuclear Factor Kappa B (NF-κB) and Interferon Response Factor 3 (IRF3), ultimately leads to the transcription of a repertoire of inflammatory cytokines characterized by a type I Interferon signature (12, 35) (**Figure 1A**). NF-κB activation may also be promoted by IKKϵ, in addition to TBK1, in macrophages (36). The cGAS-STING cascade is triggered upon cytosolic exposure of foreign nucleic acid species, following pathogen infection, but also by nucleus- and mitochondria-derived self-nucleic acids that leak into the cytosol following various types of stress (2, 37–40) and through DNA recombination processes (41).

Yet, the cGAS-cGAMP-STING signaling axis has recently emerged as far more complex than initially anticipated. First, multiple post-translational modifications influence signaling output (42–44). Second, STING can be directly activated by bacterial cyclic di-nucleotides (45, 46), while its activation is skewed by alternative di-nucleotides (16) or other metabolites (47). Third, co-sensors, co-factors and alternative upstream STING activators have been described, that can operate in cell type-specific manners (23, 48–50). Finally, in addition to the cell-autonomous capacity of cGAMP to activate STING-dependent Interferon responses, cGAS-STING signaling may also be amplified through transfer of cGAMP to neighboring cells through gap junctions (51–53), direct secretion (54) or in vesicles (55).

Below, we focus on IFI16 and DNA-PK as alternative sensors involved in the regulation of STING-dependent Interferon responses.

### IFI16: An Alternative Nucleic Acid Sensor

IFI16 is a predominantly nuclear protein that has been described as involved in the induction of innate immune responses upon infection by viruses, including Herpes simplex virus (9), Epstein-Barr virus (56), and Kaposi’s sarcoma-associated Herpes virus (57). Indeed, in this context, IFI16 promotes IRF3- and NF-κB-dependent Interferon production *via* STING (9) (**Figure 1B**). Similar to cGAS, IFI16 is capable of detecting self and non-self DNA, and displays a preference for long non-self dsDNA (58). Unlike cGAS, IFI16 operates mostly in a cell-type-dependent manner (23, 50). The interplay between cGAS and IFI16 has been explored, revealing cooperation between IFI16 and cGAS upon infection (**Figure 1B**, left). This cooperation relies on cell-type specific molecular mechanisms. Indeed, in both keratinocytes and macrophages, IFI16 cooperates with cGAS for STING activation upon infection (23, 50). However, in macrophages, IFI16 enhances cGAS-dependent cGAMP production (50), while in keratinocytes, IFI16 does not influence cGAMP production, but rather directly activates STING (23). Additionally, IFI16 has been shown to promote inflammasome activation in the nucleus, leading to production of Interleukin-1β (IL-1β), IL-18, and IL-33 cytokines (56, 59).

In contrast, following genotoxic stress, IFI16 triggers cGAS-independent STING activation (**Figure 1B**, right). Indeed, upon etoposide-induced DNA lesions, IFI16, together with DDR proteins, activates STING, promoting the assembly of a non-canonical STING signalosome (22). Within this complex IFI16 promotes TNF Receptor Associated Factor 6 (TRAF6)-dependent STING ubiquitination and activation ultimately leading to the predominant activation of the transcription factor NFκB, rather than IRF3 (22). Therefore, this signaling cascade results in the expression of a repertoire of cytokines that differs from that triggered upon cGAS-mediated detection of dsDNA, including a specific IL-6 and CCL20 signature (22). Yet, most of the described mechanisms were inferred from the study of keratinocytes or myeloid cell lines, leaving uncertainties concerning the activation of IFI16 in cancer cells.

### DNA-PK: Bridging DNA Repair and Nucleic Acid Immunity

The DNA-PK complex has been reported to play a role in controlling nucleic acid-dependent inflammation. The DNA-PK complex is a key holoenzyme, composed of KU70^XRCC6^, KU80^XRCC5^ and the DNA-dependent protein kinase catalytic subunit DNA-PKcs^PRKDC^, central to the repair of DSBs by NHEJ. NHEJ is involved in the repair of approximately 80% of DSBs and can operate regardless of the cell cycle phase. It promotes relegation of DNA ends without requirement for an intact template (60). KU70/KU80 heterodimers interact directly with damaged DNA ends and are responsible for the recruitment of DNA-PKcs to these lesions. DNA-PKcs bears a kinase activity and promotes both DNA-PKcs autophosphorylation and the phosphorylating-activation of effector proteins required for the NHEJ process. For a complete recent view of NHEJ refer to (61).

Besides its canonical role in NHEJ while it recognizes self dsDNA, there are several reports for a central role of DNA-PK in the detection of exogenous DNA species and interference with viral life cycles (62). Subunits of the complex have been independently reported to trigger or skew inflammatory responses toward either type I or type III Interferon production in response to non-self dsDNA. Indeed, KU70 triggers DNA-dependent type III Interferon responses through activation of Interferon Regulatory Factor 1 and 7 (IRF1 and IRF7) (19, 63), independently of DNA-PKcs (19).

In contrast, recent reports indicate that the catalytic activity of DNA-PKcs is also crucial for antiviral responses. Indeed, DNA-PKcs promotes IRF3 phosphorylation following infection by DNA and RNA viruses (18, 64) (**Figure 1C**). Interaction between DNA-PKcs and the progenome of the Human Immunodeficiency Virus (HIV) retrovirus has also been shown, although the link to inflammation is unexplored (65). Interestingly, some DNA viruses have evolved proteins that counteract DNA-PKcs-dependent detection (20) while others hijack NHEJ to the benefit of their replication (66), highlighting the tight interplay between viral life cycles and DDR (67). Yet, there is as of today, limited knowledge concerning the ways in which DNA-PK-dependent inflammatory responses, IFI16- and cGAS-dependent STING activation are orchestrated.

Indeed, whether DNA-PK requires STING for production of Interferons remains debated (18, 20, 68) (**Figure 1C**). It was reported that DNA-PKcs is recruited to dsDNA in the cytoplasm of both human and murine cells through KU80, triggering IRF3-dependent inflammatory responses (18). However, while some reports indicate that the catalytic activity of DNA-PKcs is responsible for direct activating phosphorylation of IRF3 (64), others indicate that the measured Interferon production can occur independently of the catalytic activity of DNA-PKcs (18). In this latter scenario, questions remain open concerning what would trigger IRF3 activation. It has also been proposed that DNA-PKcs would act upstream of TBK1 and IRF3 (18) and that KU70 can form a complex with STING prior to (18), or upon (19) DNA transfection. This notion was comforted by Morchikh et al., in 2017, showing that DNA-PK subunits (DNA-PKcs, KU70 and KU80) are associated with a ribonuclear complex that is remodeled by foreign DNA, leading to enhanced recruitment of STING, activated DNA-PKcs, and IRF3 (68). However, a recent study has shown that DNA-PKcs can also operate independently of STING (20) and that DNA-PKcs can phosphorylate cGAS and suppresses its enzymatic activity (21)(**Figure 1C**). Considering the tight link between DNA-PK activation and cell cycle progression (69), and in view of the recent reports linking cGAS activation and cell cycle stage (70), the crosstalk between DNA-PK and cGAS-STING activation would certainly benefit from integrating the temporality of events to the study. In agreement, it was previously reported that inhibition of NHEJ components reduces Interferon signaling, in a cell cycle progression-dependent manner (71).

Adding a layer of complexity, DNA-PKcs immune signaling appears to be species-specific. Indeed DNA-PKcs can activate innate immune responses independently of STING in human cells, but not in murine cells (20). This is consistent with previous reports that in mouse cell lines, where the immune response is largely dependent on STING, DNA-PKcs would signal through STING (18). Furthermore, the current state-of-the-art does not allow determining whether the role of DNA-PK in inducing type I Interferon responses may be subjected to cell type-specific regulatory mechanisms, as was reported for IFI16. In this respect, how IFI16 activation is regulated in contexts where DNA-PKcs activates inflammatory responses remains to be elucidated.

## Regulation of the DNA Damage Response by Innate Immune Sensing Pathways

### cGAS Suppresses DNA Damage Responses

**Figure 2:**
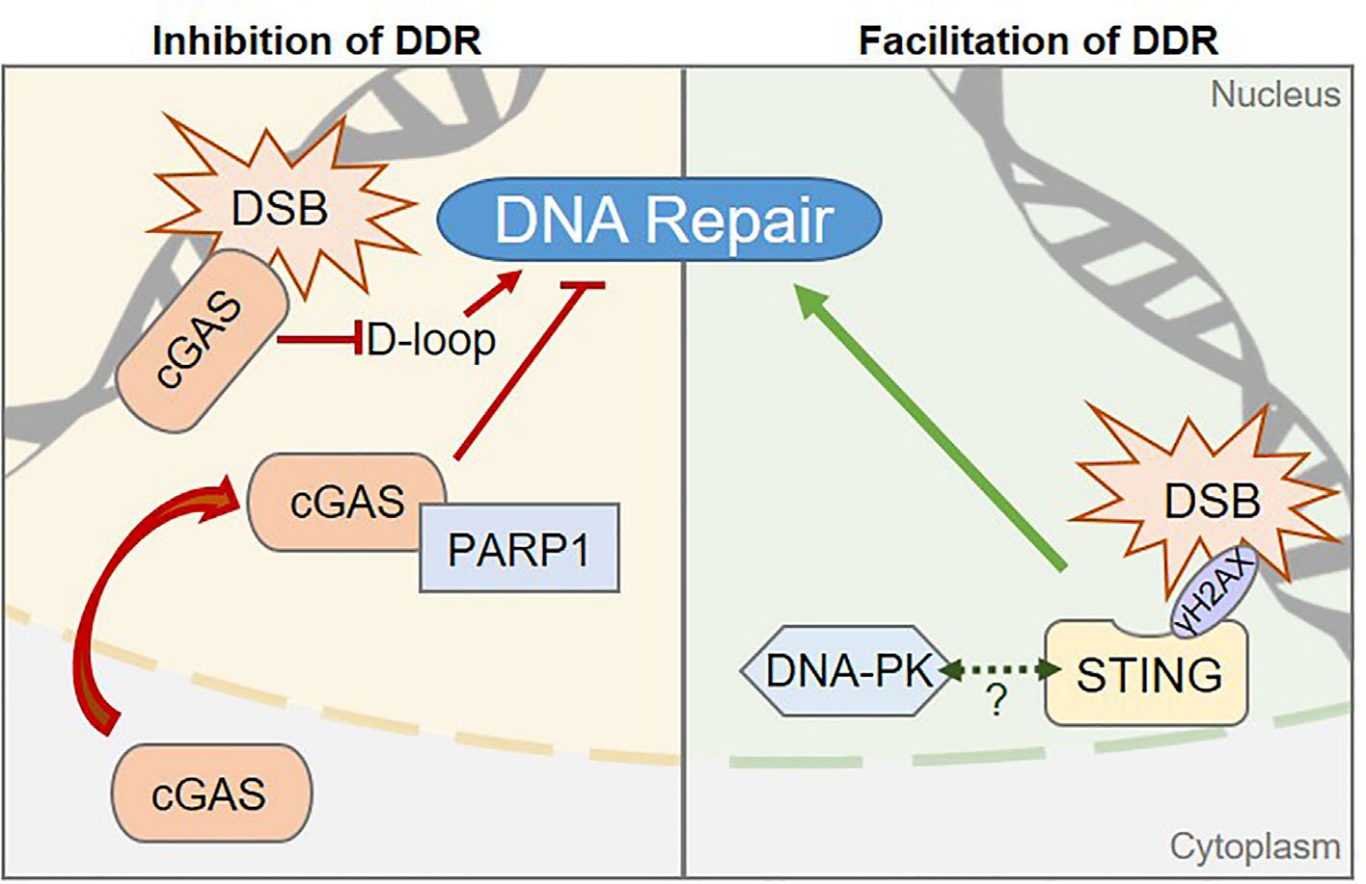
Pattern Recognition Receptors are involved in regulating DNA damage repair processes. *Left*, The cGAS sensor can inhibit DNA Damage Responses (DDR) *via* two distinct mechanisms in human cells: 1) cGAS inhibits the Homologous Recombination (HR) pathway by preventing displacement loop (D-loop) formation. 2) cGAS-PARP1 interaction impedes the formation of a PARP1-based complex required for HR-mediated DNA repair. *Right*, STING promotes DDR in human cells through a yet to be elucidated molecular mechanism, but may rely on the control of components of the Non-Homologous End Joining DNA repair pathway, such as the DNA-PK complex. Dashed arrow between STING and DNA-PK represents the reported interaction between the two proteins (19, 68, 78). Whether this interaction is related to STING-associated DDR control is unknown.

The cGAS protein was initially identified as the main receptor for cytosolic nucleic acid moieties that promote type I Interferon responses (14). However, it was recently demonstrated that an abundant pool of cGAS is tethered to the chromatin, in absence of inflammatory stimulus (72–74). Active export of cGAS through the Chromosomal Maintenance 1 (CRM1) exportin was recently demonstrated, suggesting that shuttling of cGAS to the cytosol may be a prerequisite for its activation (75). However, the molecular mechanisms triggering cGAS nuclear export and whether cGAS may also be activated in the nucleus, remains to be clarified. There is evidence for a role of cGAS in the inhibition of Homologous Recombination (HR)-mediated repair of DSB. Contrary to NHEJ, that operates in a cell cycle stage-independent manner, HR requires the presence of the sister chromatid for accurate repair of DNA lesions (76) and therefore operates mostly during the G2/M phase of the cell cycle. HR is a complex, multistep process that can be completed through several interconnected pathways, for which a complete overview can be found in (77). Two mechanisms have been proposed for cGAS-dependent HR inhibition (**Figure 2**, left). On one hand, Liu et al. showed that DNA damage triggers cGAS nuclear translocation and interaction with activated DNA damage-dependent Poly [ADP-ribose] polymerase 1 (PARP1), which is a first responder in DNA damage detection. Interaction of cGAS with PARP1 prevents the recruitment of proteins required to proceed through HR process and does not require the cGAS DNA-binding domain (79). However, since the majority of cellular cGAS is nuclear (72–74), one may question why the cytosolic rather than the chromatinian pool of cGAS would be mobilized. This may be linked to the high affinity of cGAS for the acidic patch of histones that renders chromatinian cGAS not easily displaceable (73). On the other hand, Jiang et al. observed that the DNA-binding domain of chromatin-bound cGAS is crucial for cGAS oligomerization on DNA, hindering the formation of displacement loops, which are required for HR to proceed (80). Consequently, upon irradiation, cells expressing cGAS present increased accumulation of DSBs as compared to cells that do not express cGAS. Intriguingly, this function is reportedly independent of cGAS-mediated innate immune sensing (80).

### STING as a Promoter of DNA Damage Responses

STING, the major downstream partner of cGAS, has been proposed to promote DDR and to enable cell survival, in an inflammation-independent manner (**Figure 2**, right). An important part of the regulation of STING activation is linked to its subcellular localization, with inactive STING resting in the ER and activation promoting its relocalization to the Golgi apparatus. Interestingly, in certain contexts, such as following chemotherapy regimens, STING colocalizes with γH2AX-positive DNA damage foci, at the inner nuclear membrane (78). In addition, cells knocked-down for STING present accumulation of DNA damage as compared to WT cells (78). No clear molecular mechanism has been proposed yet, although STING has been demonstrated to interact with NHEJ proteins, including DNA-PKcs, KU70 and KU80 (18, 19, 68, 78), suggesting that it may participate directly in the regulation of NHEJ. Moreover, STING overexpression leads to increased binding of DNA-PK to chromatin, suggesting that STING may cooperate with DNA-PK to control NHEJ-mediated DNA repair (78). However, the contribution of STING to NHEJ efficiency was not addressed, calling for further investigation.

Altogether, the subcellular localization of PRRs is central to the regulation of their activity, and determines whether they mediate repair- or immune-related functions. This is similar to what is witnessed for components of DNA-PK that are engaged in DNA repair or innate immune activation, depending on their subcellular localization and interactors. How these pathways cooperate or antagonize each other in given pathological situations, and in particular in the case of genotoxic stress that induces both repair and immune activation, remains to be elucidated.

## Cooperation Between DDR and Innate Immunity in Tumorigenesis

The interplay between innate immune activation and DNA repair pathways is likely to be central to our understanding of several human pathologies. For instance, several cancer susceptibility syndromes, such as Fanconi Anemia, that present with inheritable deficiencies in DNA repair pathways also display hematological disorders, such as bone marrow failure or auto-immunity, together with elevated type I Interferon levels. Mutations in DNA repair proteins are also found in diseases primarily defined as auto-inflammatory as described thoroughly in Ragu et al. (26). Indeed, deficient DDR frequently leads to pathologies, such as Ataxia-Telangiectasia, Werner Syndrome and Bloom Syndrome, in which inflammation plays a great part (81–83). Likewise, chronic inflammation plays an important role in all stages of sporadic cancer, from the onset of neoplastic lesions to metastatic dissemination (84).

Although STING targeting immunotherapies have seen a huge biomedical interest in recent years, the study of the impact of STING activation on tumorigenesis has revealed an extremely complex relationship with tumor fate. In many cases STING activation has been shown to promote tumor clearance. Nucleic acid substrates for cGAS in tumors can result from the release of chromatin fragments in the cytosol of tumor cells (85), leading to cGAS-STING activation and cell cycle arrest (85–87). In addition, released self-DNA, from dying tumor cells in the tumor microenvironment can be engulfed by intra-tumoral antigen-presenting cells (APCs), such as dendritic cells and macrophages and likely activates the cGAS-STING pathway (88), through mechanisms that are still under debate (89). The resulting cGAS-STING pathway activation promotes maturation and cross-presentation (90), ultimately leading to the recruitment and the infiltration of cytotoxic CD8(+) T cells at the tumor site (91, 92). Moreover, tumor-derived cGAMP promotes immune cells infiltration (52). Importantly, the cGAS-STING pathway was shown to potentiate the response to radiotherapy and chemotherapy (88, 93) and to synergize with checkpoint inhibitors (94, 95). Thus, activating the cGAS-STING axis in combination with chemotherapy and/or immunotherapy appears as a valuable therapeutic strategy.

However, there is evidence that cGAS-STING-dependent inflammation can fuel tumorigenesis (96), promote tolerogenic responses, impair the establishment of long-term immunity (97) and lead to chemoresistance (98, 99). Indeed, transfer of cGAMP from metastatic cells to astrocytes through gap junctions was also shown to support metastatic dissemination and chemoresistance (100). Finally, accumulation of micronuclei in the cytoplasm of cancer cells following ionizing radiation promotes STING-dependent inflammation (40, 71) and metastasis (101). It has been proposed that tumor grade and origin may account for these differential outcomes following cGAS-STING stimulation, calling for stratification strategies to identify patients that would benefit from cGAS-STING targeting immunotherapies.

Moreover, present therapeutic regimens include the use of DDR inhibitors in combination or not with radiotherapy (94, 102–104). Indeed, this approach induces accumulation of inflammatory cytosolic nucleic acids, leading to cGAS-STING pathway activation (95, 105) and promoting T cell infiltration and thus tumor regression (95, 102). Significant tumor regression has also been observed using DNA-PKcs inhibitors in combination with chemotherapy or radiotherapy (69), however the role of inflammation in this process is at present unexplored. Considering the emerging role of DDR proteins in innate immune responses, it is tempting to speculate that upon genotoxic stress, DDR proteins may directly fuel cancer-related inflammatory responses. In addition, numerous tumors down regulate the expression of cGAS and/or STING (106, 107). In these contexts, it would be important to examine if DDR proteins may take over the production of inflammatory cytokines.

Furthermore, STING activation has been shown to promote two distinct transcriptional programs. On one hand, activation of genes under the control IRF-3, leads mostly to the production of type I Interferons that are generally accepted as acting anti-cancer agents (108), while NF-κB activation promotes the production of cytokines that are mostly considered pro-tumorigenic, such as IL-6 (109, 110). Indeed, increased plasma levels of IL-6 generally negatively correlate with patient survival in many cancers (110). It would be crucial to determine whether the differential outcomes of STING activation observed in studies describing STING activation as pro-tumorigenic would result from IL-6 secretion. Ultimately, it would be crucial to determine, in those contexts where alternative receptors to cGAS would potentiate STING-dependent signaling, whether they would lead to skewing of the response toward IL-6 production and promote pathological outcomes.

Reciprocally, regulation of DDR by PRRs is likely to affect tumorigenesis. HR inhibition by chromatin-bound cGAS accelerates genome destabilization and micronuclei generation, leading to cell death both *in vitro* and *in vivo* (80). Thus, cGAS may thereby restrict the propagation of cancer cells. To the contrary, alterations of cGAS shuttling toward the cytosol correlate with poor patient prognosis (79). This suggests that nuclear translocation of cGAS and subsequent HR inhibition may promote tumorigenesis (79), although this may also be linked to defective cGAS-dependent Interferon responses. Furthermore, IFI16 has also been reported to present nuclear functions (57, 111), including a role in regulation of cell cycle arrest (111, 112). Supporting an association between IFI16 and tumorigenesis, IFI16 levels are frequently decreased in breast cancer cell lines (113). Yet, there is as of today no clear implication of IFI16-dependent cytokine production in tumorigenesis. This leaves open the possibility that IFI16 may be mobilized in tumors where cGAS expression is downregulated. Thus, deciphering the molecular cues leading to the mobilization of the different pools of cGAS, or alternative receptors such as IFI16, to detect immune-stimulatory DNA - and the impact of the different PRRs in DNA damage responses - is likely primordial to the understanding of how nucleic acid detection dictates tumor fate.

## Discussion

Accumulation of cytosolic nucleic acids, including ssDNA, dsDNA and RNA : DNA hybrids, has been documented in several etiologically distinct human pathologies that present with pathological type I Interferon responses (114). Importantly, the range of symptoms experienced by patients is broad, and as of today not fully understood.

Much attention was brought to the cGAS-STING axis, notably because it was shown that cGAS is non-dispensable for STING activation *in vivo*. Indeed, in cells, including dendritic cells, macrophages or fibroblasts, from cGAS-deficient mice, nucleic acid-dependent STING activation was abolished (115). Yet, recent research has underlined the existence of species-specificities in innate immune detection of nucleic acids (116). Thus, although the cGAS-STING cascade represents a crucial cytosolic dsDNA detection route, a more complex picture is currently emerging. In addition to the many direct regulators of the cGAS-STING pathway, alternative receptors such as IFI16 and DNA-PK, may mediate stimulus-specific Interferon responses. Therefore, previously overlooked nucleic acid sensors should be re-examined (117). In particular, the recently uncovered cooperation between DDR and nucleic acid immunity can be expected to contribute to the health alterations witnessed in patients presenting with chronic inflammation while feeding cancer susceptibility directly.

Importantly, in inflammatory pathologies, it is generally considered a risky approach to directly act on pathways responsible for Interferon production (118). This is intrinsically linked to the duality of the impacts of Interferons, that can either be beneficial or promote cytopathic effects, depending on multiple parameters that are as of today poorly understood. Several chronic inflammatory pathologies, presenting with type I Interferon overproduction, such as type I Interferonopathies, or Aicardi-Goutières Syndromes are treated with inhibitors of the Janus kinase 1, 2 and 3 (119). This treatment, rather that halting Interferon production, prevents the induction of ISGs following the interaction of Interferons with its cognate receptor. However, such disruption of immune pathways comes at the expense of increased risk of infection (119). Identification of pathways responsible for activation of pathological immune responses and the design of specific targeting strategies may be valuable in these pathologies. Addressing whether DDR proteins are involved in the inflammatory signature present in these diseases is thus important.

Altogether, the current state-of-the-art supports that STING is an attractive target for the treatment of autoimmune, inflammatory diseases and cancer (17, 120). However, emerging regulators, cell type specific or stimulus specific responses, together with alternative functions of STING and its activators, indicate that our understanding of nucleic acid immunity is still in its infancy. Our view of how immune-stimulatory DNAs are detected is likely grow in complexity, notably with the addition of DNA repair proteins to the list of PRRs. Therefore, the regulatory mechanisms and crosstalk between engaged pathways will surely remain an area of intense research in coming years.

## Author Contributions

CT, AS, IV, HC, JM, and NL drafted and edited the manuscript. Visualization: IV. All authors contributed to the article and approved the submitted version.

## Funding

Work in NL’s laboratory is supported by grants from the European Research Council (ERC-Stg CrIC: 637763, ERC-PoC DIM-CrIC: 893772), “LA LIGUE pour la recherche contre le cancer” and the “Agence Nationale de Recherche sur le SIDA et les Hépatites virales” (ANRS). CT was supported by Merck Sharp and Dohme Avenir (MSD-Avenir – GnoSTic) program, followed by an ANRS fellowship (ECTZ119088). JM was supported by a “Conventions Industrielles de Formation par la Recherche” (CIFRE) fellowship from the “Agence Nationale de Recherche Technologie” (ANRT). AS is supported by the ERC-PoC DIM-CrIC (893772). IV was supported by the European Research Council (637763) followed by the Prix Roger PROPICE pour la recherche sur le cancer du pancréas of the Fondation pour la Recherche Médicale (FRM, ARF20170938586). HC is supported by a PhD Fellowship from “La Ligue contre le cancer” (TAGQ21108). We acknowledge the SIRIC Montpellier Cancer Grant INCa_Inserm_DGOS_12553 for support.

## Conflict of Interest

JM was employed by Azelead.

The remaining authors declare that the research was conducted in the absence of any commercial or financial relationships that could be construed as a potential conflict of interest.
